# Influence of Strengthening Elements and Heat Treatment on Microstructure and Fracture Toughness of NiAl-Cr(Mo)-Based Eutectic Alloy

**DOI:** 10.3390/ma16093362

**Published:** 2023-04-25

**Authors:** Qiaoli Wang, Rui Li, Weixin Xie, Fang Yang, Beining Du, Liyuan Sheng

**Affiliations:** 1 Shenzhen Institute, Peking University, Shenzhen 518057, China; wangqiaoli@163.com (Q.W.); bndu10s@alum.imr.ac.cn (B.D.); 2 PKU-HKUST Shenzhen-HongKong Institution, Shenzhen 518057, China; lirui19930327@163.com; 3 Huizhou Port Customs, Huizhou 516081, China; xieweixin-0532@163.com; 4 Shenzhen Airlines, Shenzhen Bao’an International Airport, Shenzhen 518128, China; daisy_fangyang@163.com

**Keywords:** heat treatment, microstructure, NiAl/Cr(Mo)-based eutectic alloy, fracture toughness

## Abstract

Due to their potential improvement of high-temperature properties, the refractory metal hafnium (Hf) and the rare earth holmium (Ho) have attracted much attention. In the present research, NiAl-Cr(Mo) eutectic alloys with different Ho and Hf additions were fabricated by conventional smelting method and heat-treated to study the synergetic influence of strengthening elements and heat treatment. The samples were characterized using XRD, SEM, and TEM, and the three-point bending test was performed to obtain fracture toughness. The results demonstrate that Hf addition leads to the formation of Ni_2_AlHf Heusler phase and that Ho promoted the formation of Ni_2_Al_3_Ho phase. The microstructure of the alloy is affected by thermal treatment, with the coarsening of eutectic lamellae after heat treatment. The mechanical properties are improved by Hf and Ho additions, with increased fracture toughness. Overall, this study provides insights into the microstructure and properties of NiAl-Cr(Mo) eutectic alloys and highlights the potential of Hf and Ho addition to improve room-temperature properties. Specifically, the as-cast NiAl-Cr(Mo)-Hf eutectic alloy contains a relatively fine NiAl/Cr(Mo) eutectic lamella but coarse eutectic cell with Ni_2_AlHf phase embellished along the cell boundary. Minor Ho addition induces the formation of Ni_2_Al_3_Ho phase, which leads to the coarsening of the intercellular region but contributes to the refining of eutectic cell. In addition, the synergetic effect of Ho and Hf promotes the precipitation of Ni_2_Al_3_Ho and Ni_2_AlHf phases in the intercellular zone and increases the interface dislocations. Heat treatment benefits the solid solution of Ni_2_Al_3_Ho and Ni_2_AlHf phases, which improves their size and distribution by secondary precipitation. The Ni_2_AlHf phase in the NiAl-Cr(Mo)-Hf eutectic alloy becomes fine and uniformly distributed, but the NiAl/Cr(Mo) eutectic lamella in the eutectic cell becomes coarse. In comparison, heat treatment mainly optimizes the size and distribution of the Ni_2_Al_3_Ho and Ni_2_AlHf phases in the NiAl-Cr(Mo)-Hf-Ho eutectic alloy. Furthermore, heat treatment helps to eliminate the interface dislocations in the large NiAl precipitates and the NiAl/Cr(Mo) phase interfaces, which also contributes to fracture toughness by decreasing stress concentration. Minor Ho addition decreases the fracture toughness of as-cast NiAl-Cr(Mo)-Hf eutectic alloy from 6.7 to 6.1 MPa·m^1/2^, which should be ascribed to the coarsened intercellular region including aggregated Ni_2_Al_3_Ho and Ni_2_AlHf phases. However, minor Ho-doped NiAl-Cr(Mo)-Hf eutectic alloy obtained the highest fracture toughness of 8.2 MPa·m^1/2^ after heat treatment. This improved fracture toughness should be mainly attributed to the refined and well-distributed Ni_2_Al_3_Ho and Ni_2_AlHf phases in the heat-treated NiAl-Cr(Mo)-Hf-Ho eutectic alloy.

## 1. Introduction

Intermetallic compounds have attracted significant attention due to their special interfacial chemical properties, high melting point, high specific strength, and good environment tolerance [1,2,3,4,5]. As one kind of intermetallic compound, NiAl possesses many attractive advantages, such as a relatively low density, excellent corrosion and oxidation resistance, high melting point, and excellent thermal conductivity [6,7,8,9]. Hence, it has been thought to be a potential candidate for high-temperature structural materials that can endure severe oxidation and corrosion well [10,11]. Based on the previous research [1,12], the attractive properties of NiAl should be ascribed to its long-range ordered crystal structure, but this feature also results in its brittleness at low temperature. Though NiAl demonstrates well-improved ductility at high temperature, its mechanical properties still cannot meet the requirements of structural parts with load at room temperature [13,14]. Nevertheless, the outstanding oxidation and corrosion resistance of NiAl makes it very attractive as a fixed part or protective shield [15]. Regardless, these applications also present requirements for NiAl-based materials, including room-temperature strength (800 MPa) and fracture toughness (18 MPa·m^1/2^) [6,10,12]. Therefore, it is still a critical research issue to simultaneously enhance the fracture toughness and strength of NiAl-based materials.

To improve the fracture toughness and strength of NiAl-based materials, many techniques have been tried and many NiAl-based alloys or composites have been designed and fabricated [16,17,18,19]. Among these NiAl-based materials, the ceramic-particle-strengthened NiAl-based composites exhibit high strength, while the fiber- or lamella-phase-strengthened alloys demonstrate better fracture toughness [20,21,22]. Hu et al. [23] fabricated Ni_3_(Al,Ti)- and TiC-strengthened NiAl-based composite and demonstrated that the composite had ultra-high hardness and increased fracture toughness when the temperature was below 800 °C. The cleavage in the binder NiAl compound and de-cohesion of TiC from the Ni_3_(Al,Ti) interface were determined as the dominating fracture mechanism. However, the ductility of this kind of composite is much lower, and there have been few reports on it. Compared with the ceramic-strengthened NiAl-based composite, the lamellar- or fiber-phase-strengthened NiAl-based alloys demonstrated more advantages in ductility. Yang et al. [24] prepared NiAl/Cr(Mo) eutectic alloy and studied the deformation mechanism, which indicated that the appropriate NiAl and Cr(Mo) lamellar structure was important for mechanical properties. To further improve the high-temperature creep properties, Hu et al. [21] fabricated a fiber-reinforced NiAl-9Mo eutectic alloy and revealed that the tensile strength and creep properties could be enhanced significantly, which was mainly attributed to the well-aligned Mo fiber phases. However, compared with a superalloy, the high-temperature and low-temperature mechanical properties of the lamellar- or fiber-phase-strengthened NiAl-based eutectic alloy still need further improvement [25,26,27,28]. To improve the strength of NiAl-based eutectic alloy, in situ or extrinsic strengthening phases are always the preferred methods, due to their effect and convenience. Previous research [29,30,31] has shown that the introduction of Laves phases in NiAl/Cr(Mo) eutectic alloy improves its strength, but this kind of strengthening phase prefers to aggregate in intercellular zones and reduces compressive ductility. Research [32,33,34] on NiAl/Cr(Mo) eutectic alloy with the addition of Hf also revealed that Heusler phase could improve the strength noticeably, but its segregation along the boundary is apt to induce cracks and decrease the deformability. Therefore, how to optimize the microstructure and strengthening phase distribution for NiAl-based eutectic alloy has become a critical issue to increase its mechanical properties further. Guo et al. [35] had revealed that a minor addition of Ho could contribute to microstructure refinement and increase the mechanical properties. Moreover, a recent investigation [36] has demonstrated that heat treatment can improve the distribution and morphology of the strengthening phase and contribute to compressive ductility. However, the interaction between the strengthening alloy elements and heat treatment may exert diverse effects on the microstructure and mechanical properties. Therefore, it is necessary to clarify the integrated effect and internal mechanism, in order to obtain further improvement in the mechanical properties.

Based on the analyses above, minor Hf-doped NiAl-Cr(Mo) eutectic alloy was chosen in the present research. To optimize the distribution of Hf and its strengthening phase, minor Ho was added to the alloy. The two-stage heat treatment was performed on the NiAl-Cr(Mo) eutectic alloy with different Ho and Hf additions. The microstructure, phase constituent, and fracture toughness were analyzed to reveal the synergistic influence of heat treatment and strengthening element on microstructure evolution and mechanical properties.

## 2. Materials and Methods

Pure nickel (99.9%), chromium (99.9%), hafnium (99.8%), holmium (99.8%), aluminum (99.9%), and molybdenum (99.8%) were used to fabricate the different Ho- and Hf-doped NiAl-Cr(Mo) eutectic alloys, with nominal compositions of Ni-33Al-28Cr-5Mo-0.2Hf and Ni-33Al-28Cr-5Mo-0.2Hf-0.2Ho (at.%), by vacuum induction melting. During the smelting of alloys, the nickel, chromium, aluminum, and molybdenum were first remelted, and then the hafnium and holmium enfolded by nickel foil were inserted into the melt. After this, the melt was continuously heated for 20 min to ensure a homogeneous composition. The molten alloy liquid was poured into a ceramic shell preheated to 973 K to obtain as-cast rods (φ50 mm × 200 mm). These as-cast rods were cut off and divided into two groups. One group was studied in its as-cast state, and the other group was heat treated. The two-stage heat treatment included 1473 K for 2 h and 1273 K for 24 h, subsequently followed by furnace cooling. The cooling rate was approximately 10 K/min.

**Figure 1:**
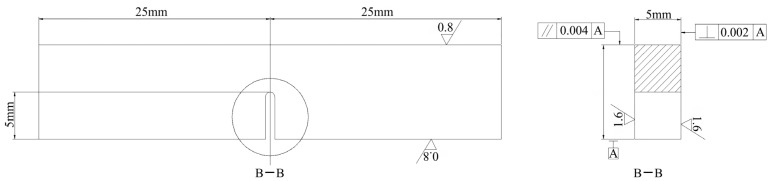
The dimensions of the bending test specimen.

Specimens for microstructure analysis and fracture toughness test were prepared from the alloys with different states. All specimens were processed by the conventional metallographic method. S-3400 and Phenom™ Pro scanning electron microscopes (SEMs) were applied to perform the microstructure observation. The crystallographic phase and preferential orientations of different specimens were analyzed by Bruker D2 X-ray diffractometer (XRD) with a copper Kα radiation source. EPMA-1610 electronic probe microanalysis (EPMA) was used to analyze the chemical compositions of different phases in the as-cast alloys. The observations of crystal defects and precipitates were carried out on a JEOL-2010 transmission electron microscope (TEM). Slices of 0.4 mm thickness were cut from alloys of different states and polished to 30 μm. Then, these slices were shaped into φ3 mm pieces. The shaped slices were continuous processed by twin-jet electropolishing in a solution of 10% perchloric acid and 90% alcohol at 253 K. The twin-jet electropolishing current was kept at 40 mA. Based on the GBT 4161-2007 standard, the three-point bending test was carried out to obtain the fracture toughness. The specimens for the bending test, with a size of 5 mm × 10 mm × 50 mm and a 5 mm notch in the middle, were cut from the alloys of different states, as exhibited in Figure 1. The test was performed at room temperature, and four specimens were tested for each alloy. After the bending test, the fracture surfaces were observed to analyze the crack propagation.

## 3. Results and Discussion

### 3.1. Microstructure Characteristic

**Figure 2:**
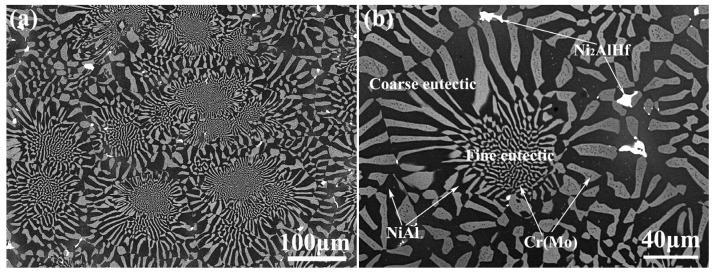
SEM micrograph of the as-cast NiAl-Cr(Mo)-Hf eutectic alloy: (**a**) microstructure of near eutectic structure and precipitates; (**b**) morphology of fine and coarse eutectic structure in eutectic cell.

SEM observations were performed on the as-cast NiAl-Cr(Mo)-Hf eutectic alloy, and the results are demonstrated in Figure 2. It clearly demonstrates the chrysanthemum-like microstructure with central fine lamella and peripheral coarse eutectic lamella, as exhibited in Figure 2a. The gray Cr(Mo) phase and black NiAl phase comprise the NiAl/Cr(Mo) eutectic structure. In the center of the eutectic cell, the NiAl and Cr(Mo) phases have relatively fine lamellae, and they becomes coarse as they extend to the eutectic cell boundary, as exhibited in Figure 2b. Moreover, some white phases could be observed along the cell boundary. The chemical composition analyses of the phases indicate that the NiAl phase has a small content of Cr, while the Cr(Mo) phase has some Ni and Al, as exhibited in Table 1. Comparatively, the Cr(Mo) phase has a higher solid solubility. The white phase mainly contains Ni, Al, and Hf elements and a small content of Cr. According to past research [37,38], the white phase could be determined as Heusler phase, which has high stiffness. It can be found that there are two sizes of eutectic cell. One has a large size of about 200 μm, and the other has a small size of about 100 μm. The average thickness of the fine eutectic lamellae is about 2 μm, while that of the coarse eutectic lamellae is about 8 μm. Furthermore, many small Cr(Mo) precipitates could be observed in the NiAl phase, and there were many small NiAl precipitates in the Cr(Mo) phase.

**Figure 3:**
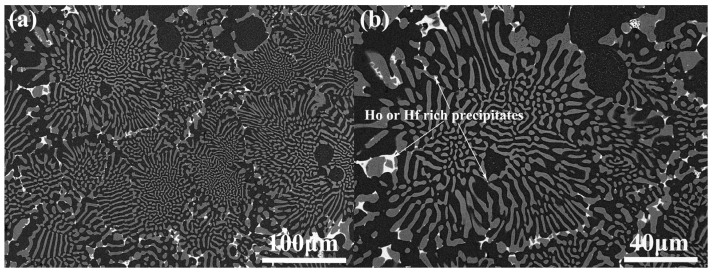
SEM micrograph of the as-cast NiAl-Cr(Mo)-Hf-Ho alloy: (**a**) microstructure of near eutectic structure with coarse NiAl phase; (**b**) morphology of semi-continuously precipitates distributed along eutectic cell boundary.

Figure 3 shows the SEM observations of the as-cast NiAl-Cr(Mo)-Hf-Ho eutectic alloy. It is found that the addition of Ho has changed the microstructure greatly. Though the alloy still has the eutectic structure, many coarse Cr(Mo) and NiAl phases have formed in the alloy, as exhibited in Figure 3a. In addition, the eutectic cell has lost the fine lamellae in the center. The relatively coarse Cr(Mo) and NiAl phases grow from the center of the eutectic cell to the boundary. Moreover, the white phases along the eutectic cell boundaries have almost formed a semi-continuous chain. The chemical composition analyses of the phases reveal that the solid solute content of the Cr(Mo) and NiAl phases decreases slightly, as shown in Table 1. Compared with the as-cast NiAl-Cr(Mo)-Hf eutectic alloy, the content of Cr in NiAl phase and Ni in Cr(Mo) phase decreases, but the Al content in Cr(Mo) phase increases slightly. The white phases could be divided into two kinds. One is rich of Ni, Al, and Hf and contains some Cr and Ho, while the other is rich of Ni, Al, Ho and contains some Cr and Hf. Based on previous research [35,39], the Hf-containing phase should be the Ni_2_AlHf Heusler phase and the Ho-rich phase could be Ni_2_Al_3_Ho phase. Observations of the precipitates in the Cr(Mo) and NiAl phase indicate that the small Ho addition has promoted the redistribution and relative coarsening of Heusler phases, as exhibited in Figure 3b. The statistical analysis of the size of eutectic lamellae and cells reveals that the average size of the eutectic lamellae is about 5 μm, while that of eutectic cells is about 100 μm. Compared with the NiAl-Cr(Mo)-Hf eutectic alloy, the addition of Ho results in the refinement of the eutectic cell. However, the presence of Ho also leads to the coarse intercellular region and NiAl/Cr(Mo) eutectic phases.

**Figure 4:**
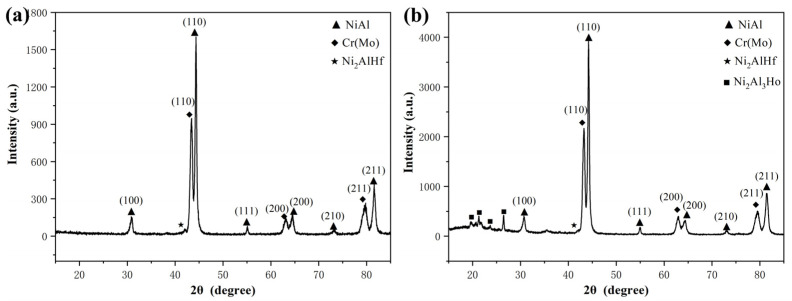
XRD patterns of the as-cast eutectic alloys: (**a**) NiAl-Cr(Mo)-Hf alloy; (**b**) NiAl-Cr(Mo)-Hf-Ho alloy.

To analyze the phase and crystallographic orientation in the as-cast NiAl-Cr(Mo)-Hf and NiAl-Cr(Mo)-Hf-Ho eutectic alloy, XRD analysis has been employed, and the results are given in Figure 4. Clearly, the as-cast NiAl-Cr(Mo)-Hf eutectic alloy mainly comprises NiAl, Cr(Mo), and Ni_2_AlHf phases, as exhibited in Figure 4a. The NiAl phase grows along the (100), (110), (111), (200), (210), and (211) crystallographic planes, and the (110) is the most preferred one. For the Cr(Mo) phase, it mainly grows along the (110), (200), and (211) crystallographic planes, and the (110) is the most preferred one. The minor Ho addition in the as-cast NiAl-Cr(Mo)-Hf eutectic alloy has resulted in some new diffraction peaks when the 2θ is between 20° and 30°, as exhibited in Figure 4b. Additionally, the Ho addition has strengthened the diffraction peak of Cr(Mo) along (200). Except for these small changes, the as-cast NiAl-Cr(Mo)-Hf-Ho eutectic alloy demonstrates diffraction peaks almost similar to those of the NiAl-Cr(Mo)-Hf eutectic alloy. Based on a combination of SEM observation and composition analysis, the new diffraction peaks should be the Ni_2_Al_3_Ho phase.

**Figure 5:**
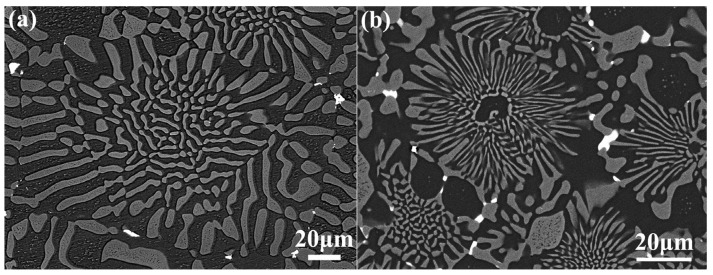
SEM micrograph of the eutectic alloys with heat treatment: (**a**) NiAl-Cr(Mo)-Hf alloy; (**b**) NiAl-Cr(Mo)-Hf-Ho alloy.

The SEM observations of the NiAl-Cr(Mo)-Hf and NiAl-Cr(Mo)-Hf-Ho eutectic alloys with two-stage heat treatment are exhibited in Figure 5. Compared with the as-cast alloy, the microstructures of heat-treated alloys have been obviously changed. After the heat treatment, the NiAl-Cr(Mo)-Hf eutectic alloy has almost lost the fine lamellar structure in the center, which is obviously coarsened, as exhibited in Figure 5a. However, the size of the NiAl/Cr(Mo) eutectic lamellae in the peripheral region of eutectic cell has almost no change, which promotes the homogeneous microstructure. The Ni_2_AlHf phases still prefer to precipitate along the eutectic cell boundary, but they exhibit small size and homogeneous distribution. For the heat-treated NiAl-Cr(Mo)-Hf-Ho eutectic alloy, it demonstrates an obvious microstructure evolution, as exhibited in Figure 5b. Different from the as-cast NiAl-Cr(Mo)-Hf-Ho eutectic alloy, the heat treatment has changed the semi-continuously distributed Ni_2_Al_3_Ho or Ni_2_AlHf phases into separately distributed ones. Furthermore, the amount and size of Ho-rich or Ni_2_AlHf phases is decreased, and their location is still mainly in the intercellular region. What is interesting is that the heat treatment has not resulted in an obvious coarsening of the NiAl/Cr(Mo) eutectic lamellae in the eutectic cell, but the amount of coarse Cr(Mo) phase in the intercellular zone has decreased a small amount. Compared with the as-cast eutectic alloys, the quantity and size of small precipitates in the Cr(Mo) and NiAl phases in the heat-treated alloys are decreased simultaneously. In general, the heat treatment promotes the eutectic alloys to have the features of homogeneous microstructure and refined precipitates. Actually, the precipitates can play an important role in the strengthening effect. In particular, the stiffness phase, its size, morphology, and distribution would influence the ductility and strength of the alloy. The well-distributed and refined strengthening phase in the present heat-treated alloy would benefit from these mechanical properties.

**Figure 6:**
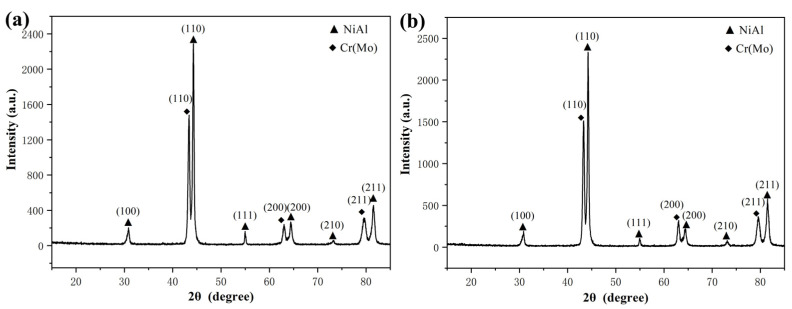
XRD patterns of the eutectic alloys with heat treatment: (**a**) NiAl-Cr(Mo)-Hf alloy; (**b**) NiAl-Cr(Mo)-Hf-Ho alloy.

Further XRD analyses on the heat-treated NiAl-Cr(Mo)-Hf and NiAl-Cr(Mo)-Hf-Ho eutectic alloys are exhibited in Figure 6. Clearly, the crystallographic orientations of the Cr(Mo) and NiAl phases in the heat-treated alloy demonstrate almost similar features as those in the as-cast alloy. The difference is that the heat treatment almost eliminates the diffraction peaks of the Ni_2_AlHf phase, and other diffraction peaks are strengthened in the NiAl-Cr(Mo)-Hf eutectic alloy, as exhibited in Figure 6a. Such a phenomenon indicates that some Ni_2_AlHf phase has been solid-soluted by heat treatment, which is in accordance with the SEM observation. For the NiAl-Cr(Mo)-Hf-Ho eutectic alloy, the heat treatment eliminates the small diffraction peaks located below 2θ of 30°, as exhibited in Figure 6b. In addition, the diffraction peak of Ni_2_AlHf has also disappeared, which indicates that the most stiff phases precipitated in the alloy have been solid-soluted by the heat treatment. The (200) crystallographic plane diffraction peak of the Cr(Mo) phase is still stronger than that of the NiAl phase, which implies that the crystallographic orientation preference effect by Ho addition still exists.

**Figure 7:**
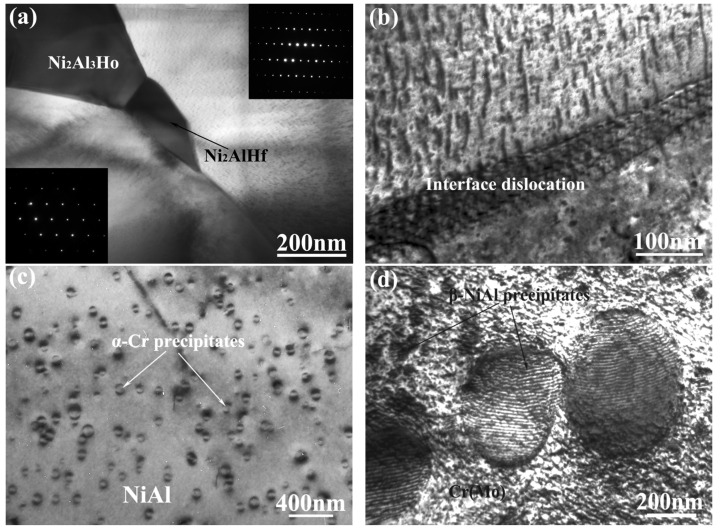
TEM observations of the as-cast NiAl-Cr(Mo)-Hf-Ho eutectic alloys: (**a**) TEM micrograph of Ni_2_AlHf and Ni_2_Al_3_Ho phase (upper-right inset image exhibits the SAED pattern of Ni_2_Al_3_Ho phase, and left-bottom inset image exhibits the SAED pattern of Ni_2_AlHf phase); (**b**) TEM micrograph of the interface dislocation along the NiAl/Cr(Mo) phase interface; (**c**) morphology of α-Cr precipitates in NiAl phase; (**d**) morphology of β-NiAl precipitates in Cr(Mo) phase.

TEM observations on the as-cast NiAl-Cr(Mo)-Hf-Ho eutectic alloys are given in Figure 7. Obviously, the Ho addition induces the precipitation of Ni_2_Al_3_Ho phase, which prefers to precipitate with the Ni_2_AlHf phase, as exhibited in Figure 7a. The corresponding selected area electron diffraction (SAED) pattern confirms that the Ho-rich phase is Ni_2_Al_3_Ho phase, having a hexagonal crystal structure with a = 0.902 mm, c = 0.4052 mm, and space group of P62/mmm. By the SAED pattern, the Ni_2_AlHf phase is confirmed to have a cubic crystal structure with a = 0.6810 nm and space group of Fm3m. Though the Ni_2_AlHf and Ni_2_Al_3_Ho phases prefer to coexist, no crystal orientation relationship between them has been obtained in the present research. Further observation on the interface between Cr(Mo) and NiAl phases shows a large number of interface dislocation networks formed, as exhibited in Figure 7b. According to past studies [12,24], Cr(Mo) and NiAl phases possess good interface matching, because they have the same crystal structure and a similar lattice constant, especially along the (100) crystallographic plane. The presence of interface dislocation indicates that there is great lattice distortion along the phase interface, which may be induced by the segregation of Ho or Hf atoms. The interface dislocation network between Cr(Mo) and NiAl phases could restrict the traversal of mobile dislocations and contribute to the strength of NiAl-Cr(Mo)-based eutectic alloy. Observations of the NiAl phase reveal the formation of small α-Cr precipitates with bean shape and 50–100 nm diameter, as exhibited in Figure 7c. Such a morphology of α-Cr precipitates indicates that there is a near-coherent interface between NiAl phase and α-Cr precipitate [40]. However, observation of the Cr(Mo) phase reveals two kinds of β-NiAl precipitates, with coarse and fine morphology, as exhibited in Figure 7d. The coarse one has a diameter of about 400 nm, with interface dislocations inside, while the fine one has a diameter of about 10 nm, with relatively uniform distribution. Based on past research [24,39], Cr(Mo) or NiAl precipitates with bean shape would possess a coherent interface, while those with interface dislocation possess an almost semi-coherent interface. It seems that the coexistence of Ho and Hf mainly influences the NiAl/Cr(Mo) phase interface and the β-NiAl precipitates in the Cr(Mo) phase, which may be ascribed to the preferential segregation of Ho or Hf along these phase interfaces.

**Figure 8:**
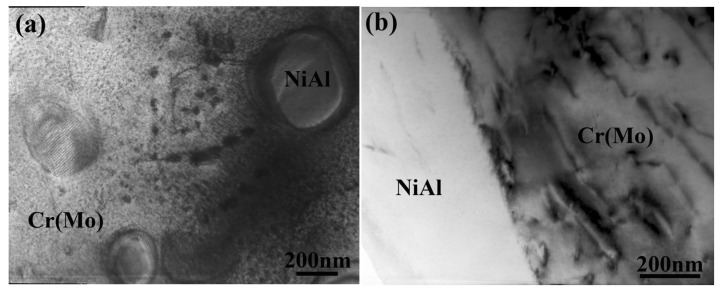
TEM observations of the heat-treated NiAl-Cr(Mo)-Hf-Ho eutectic alloys: (**a**) morphology of NiAl precipitates in Cr(Mo) phase; (**b**) morphology of NiAl/Cr(Mo) phase interface.

TEM observations of the heat-treated NiAl-Cr(Mo)-Hf-Ho eutectic alloy are exhibited in Figure 8. These demonstrate that the morphology of NiAl precipitates in the Cr(Mo) phase have experienced some changes after the heat treatment, as exhibited in Figure 8a. The interface dislocations have been eliminated in some NiAl precipitates with large size, but the interface dislocations still can be found along the phase interface. Additionally, the fine NiAl precipitates with bean morphology could be found in the Cr(Mo) phase. The TEM observations of the NiAl/Cr(Mo) phase interface reveal that the heat treatment has decreased the width of the interface dislocation, as exhibited in Figure 8b. Though the residual interface dislocation can still be observed, the straight interface is the main feature. In the Cr(Mo) and NiAl phases, there are abundant dislocations, which are tangled. This evolution of precipitate and interface morphology indicates that the heat treatment eliminates some crystal defects by the elements’ diffusion [41].

### 3.2. Mechanical Properties

**Figure 9:**
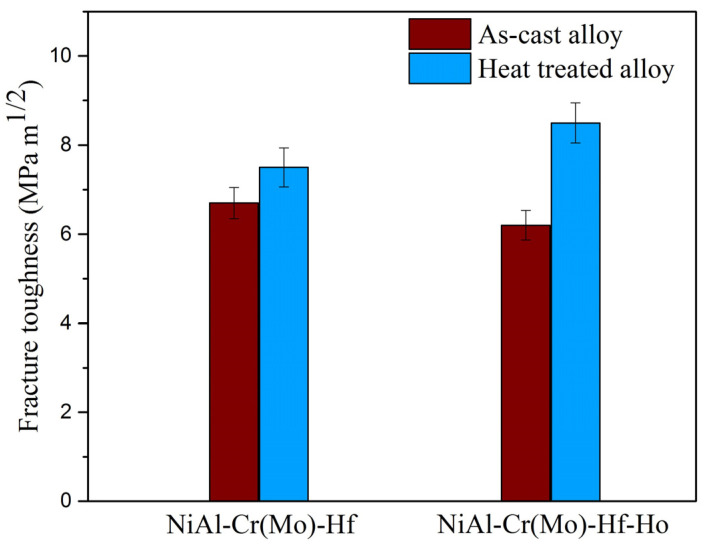
Fracture toughness of the NiAl-Cr(Mo)-Hf and NiAl-Cr(Mo)-Hf-Ho alloys in different states.

The fracture toughness of as-cast and heat-treated NiAl-Cr(Mo)-Hf and NiAl-Cr(Mo)-Hf-Ho eutectic alloys measured by three-point bending tests are exhibited in Figure 9. The results indicate that the as-cast NiAl-Cr(Mo)-Hf eutectic alloy, heat-treated NiAl-Cr(Mo)-Hf eutectic alloy, as-cast NiAl-Cr(Mo)-Hf-Ho eutectic alloy, and heat-treated NiAl-Cr(Mo)-Hf-Ho eutectic alloy have an average fracture toughness of 6.7 MPa·m^1/2^, 7.6 MPa·m^1/2^, 6.1 MPa·m^1/2^, and 8.2 MPa·m^1/2^, respectively. Compared with the as-cast NiAl-Cr(Mo)-Hf eutectic alloy, the Ho addition in the alloy has decreased the fracture toughness slightly, which should be mainly attributed to the coarsened intercellular zone and increased stiffness phases. However, compared with the heat-treated NiAl-Cr(Mo)-Hf eutectic alloy, the fracture toughness of the Ho-doped NiAl-Cr(Mo)-Hf eutectic alloy has increased slightly. In contrast, the increasing ratio of fracture toughness of the NiAl-Cr(Mo)-Hf-Ho eutectic alloy is higher than that of the NiAl-Cr(Mo)-Hf eutectic alloy. Such an evolution of fracture toughness might be ascribed to the optimized microstructure by the heat treatment, especially the size and distribution of the stiffness phases. Moreover, the elimination of crystal defects also helps to enhance fracture toughness, because of the decreasing of stress concentration.

**Figure 10:**
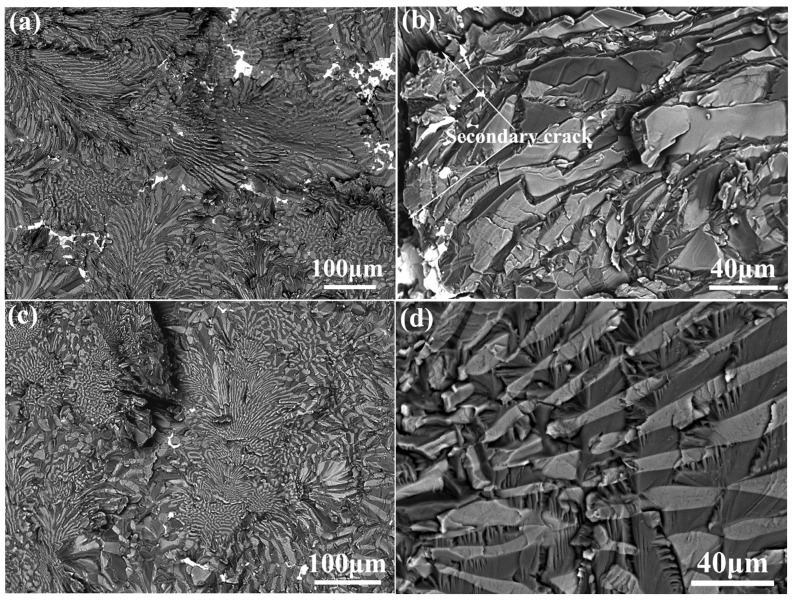
Fracture surface of the NiAl-Cr(Mo)-Hf eutectic alloy in different states: (**a**) morphology of the fracture surface of the as-cast alloy; (**b**) secondary crack and debonding along phase interface in as-cast alloy; (**c**) morphology of fracture surface of the heat-treated alloy; (**d**) morphology of the cleavage traversing a NiAl/Cr(Mo) eutectic lamella in heat-treated alloy.

To further verify the deduction on the fracture toughness evolution, the fracture surfaces of all failed specimens have been observed. For the as-cast NiAl-Cr(Mo)-Hf eutectic alloy, the cleavage is the main characteristic, as exhibited in Figure 10a,b. A brittle cleavage fracture with river patterns can be found on the fracture surface. Moreover, some secondary cracks propagating along the eutectic cell boundary could be found, which indicates that the intercellular region is relatively weaker in strength and detrimental to the fracture toughness. A cleavage-like fracture of coarse Cr(Mo) phase is also observed in the as-cast alloy, which implies that rapid crack propagation has resulted in a similar failure mode of Cr(Mo) phase with NiAl phase during fracture. In general, the relatively flat fracture surface of the as-cast NiAl-Cr(Mo)-Hf eutectic alloy indicates relatively little resistance to crack propagation, thereby resulting in a lower value of fracture toughness. For the heat-treated NiAl-Cr(Mo)-Hf eutectic alloy, transgranular fracture plays an important role, as exhibited in Figure 10c,d. Especially in the center of the eutectic cell, the NiAl/Cr(Mo) eutectic lamella is almost fractured perpendicular to its growth direction and diverges from the transgranular fracture morphology. However, in the intercellular region, it demonstrates a mixing of cleavage and transgranular fracture, which indicates the reflection or transition of cracks. Further observation on the NiAl/Cr(Mo) eutectic lamella reveals tearing ridges, indicating the fracture-energy-absorbing effect of the fine eutectic lamellar structure. Compared with the NiAl phase, the Cr(Mo) phase exhibits a better bridging effect because of its relatively high strength and ductility. Therefore, the well-aligned eutectic lamellar structure contributes much to the improvement of fracture toughness.

**Figure 11:**
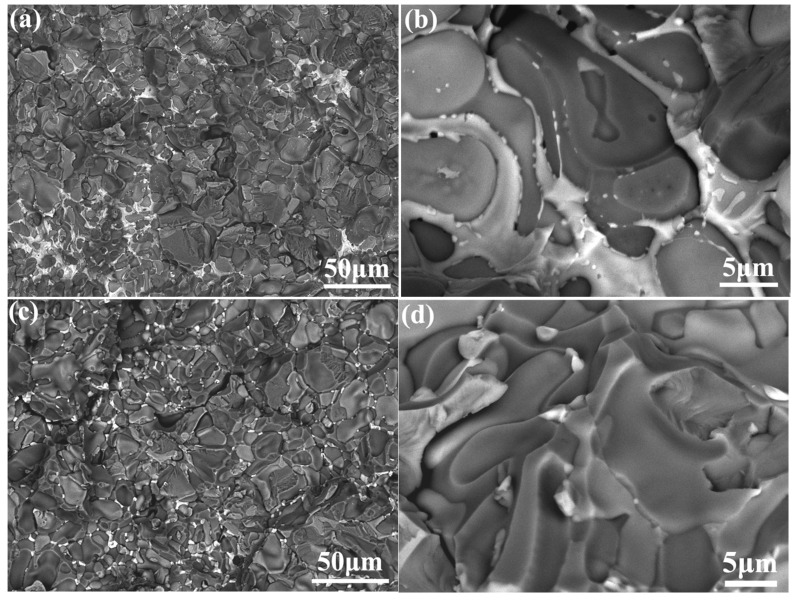
Fracture surface of the NiAl-Cr(Mo)-Hf-Ho eutectic alloy in different states: (**a**) morphology of fracture surface of the as-cast alloy; (**b**) secondary crack and debonding along phase interface in as-cast alloy; (**c**) morphology of fracture surface of the heat-treated alloy; (**d**) morphology of the cleavage traversing NiAl/Cr(Mo) eutectic lamella in heat-treated alloy.

Compared with the NiAl-Cr(Mo)-Hf eutectic alloy, the fracture surface of the NiAl-Cr(Mo)-Hf-Ho eutectic alloy demonstrates a different morphology, as exhibited in Figure 11. In the as-cast alloy, the debonding along the phase interface is the main feature, as exhibited in Figure 11a,b. Whether in the intercellular region or the eutectic cell center, the debonding plays an important role, accompanied by some cleavage. Such a phenomenon indicates that the NiAl/Cr(Mo) phase interface is the weakest location in terms of bonding strength. During the fracture, the crack would prefer propagating along the phase interface. Moreover, the white phase lining the intercellular region also demonstrates the debonding feature. Further observation of the corresponding region reveals that there are two kinds of white phases, which should be coexisting Ni_2_AlHf and Ni_2_Al_3_Ho phase. These strengthening phases also exhibit a smooth surface, which indicates that debonding is the main failure mode. In general, the ratio of cleavage in the fracture surface of the as-cast NiAl-Cr(Mo)-Hf-Ho eutectic alloy is much lower than that of debonding. The heat treatment changes the fracture surface morphology of the NiAl-Cr(Mo)-Hf-Ho eutectic alloy slightly, as exhibited in Figure 11c,d. Though a rough observation demonstrates that debonding is still the main characteristic, the magnified image reveals that there are lots of cleavages. In addition, the small precipitates of Ni_2_AlHf and Ni_2_Al_3_Ho also exhibit some transgranular fractures, which indicates these stiffness phases have acted as obstacles of cracking. The rough analyses indicate that the ratio of cleavage fracture in heat-treated alloy is slightly higher than that in as-cast alloy. Such a morphology feature could be attributed to the microstructure’s optimization by heat treatment. The more reasonable ratio of cleavage in the heat-treated alloy contributes to making full use of the ductile Cr(Mo) phase and stiffness phase. Therefore, the heat-treated NiAl-Cr(Mo)-Hf-Ho eutectic alloy possesses the highest fracture toughness.

Based on recent research [42,43,44], the mechanical properties of an alloy are closely related to its microstructure, precipitate, crystal orientation, and crystal defects. In the present study, the NiAl/Cr(Mo) eutectic lamella is the main structure, which comprises ductile Cr(Mo) phase and brittle NiAl phase. The finer lamella would make full use of the ductile Cr(Mo) phase, which reflects the cracks and handicaps related to their rapid propagation. Therefore, the refinement of microstructure could contribute most to the fracture toughness and strength. The addition of Hf could strengthen the eutectic alloy by the precipitation along the phase interface, which acts as the dislocation obstacle and contributes to the high-temperature strength. However, the large atom radius of Hf leads to its enrichment in front of the liquid/solid interface, which raises the constitutional supercooling and induces the coarsening of eutectic lamella. That is why the intercellular region contains coarse NiAl and Cr(Mo) phases. This characteristic of the NiAl-Cr(Mo)-Hf eutectic alloy during solidification results in the formation of Ni_2_AlHf phases along the phase interface. Especially in the intercellular region, the exceeding enrichment of Hf promotes the coarse Ni_2_AlHf phases, which would reduce room-temperature mechanical properties, especially ductility. Heat treatment could promote the solid solution and secondary precipitation of Ni_2_AlHf phase, but the coarse Cr(Mo) and NiAl phase cannot be changed. Therefore, the enhancement of fracture toughness of NiAl-Cr(Mo)-Hf eutectic alloy is not higher after heat treatment. The Ho addition could increase the nucleus during solidification, which benefits the microstructure refinement. The Ho would cooperate with Hf to increase the enrichment in front of the liquid/solid interface and constitutional supercooling, which coarsens the NiAl and Cr(Mo) phases in the intercellular region. In particular, the Ni_2_AlHf and Ni_2_Al_3_Ho phases in the intercellular zone become very coarse and almost continuously distributed. Therefore, the refining effect of the eutectic cell and eutectic lamella is offset by the coarsened intercellular region, which results in the lower fracture toughness of the as-cast NiAl-Cr(Mo)-Hf-Ho eutectic alloy compared to that of the as-cast NiAl-Cr(Mo)-Hf eutectic alloy. The heat treatment refines the Ni_2_Al_3_Ho and Ni_2_AlHf phases and regulates their distribution, which optimizes the microstructure and benefits the fracture toughness. Furthermore, the improved NiAl precipitates the in Cr(Mo) phase and the phase interface morphology contribute to the fracture toughness as well. However, the original coarse phase could not be changed in the subsequent heat treatment, which influences the improvement of mechanical properties. Therefore, the initial alloying composition and solidification processing should be further optimized to obtain better fracture toughness.

## 4. Conclusions

(1) The as-cast NiAl-Cr(Mo)-Hf eutectic alloy contains a relatively fine NiAl/Cr(Mo) eutectic lamella but coarse eutectic cell with Ni_2_AlHf phase embellished along the cell boundary. The minor Ho addition induces the formation of Ni_2_Al_3_Ho phase and refines the eutectic cells but coarsens the intercellular zone. In addition, the synergetic effect of Ho and Hf promotes the precipitation of Ni_2_Al_3_Ho and Ni_2_AlHf phases in the intercellular zone and increases the interface dislocations.
(2) The heat treatment facilitates the solid solution of Ni_2_Al_3_Ho and Ni_2_AlHf phases, which benefits their size and distribution by secondary precipitation. For the NiAl-Cr(Mo)-Hf eutectic alloy, its NiAl/Cr(Mo) eutectic lamella in the eutectic cell becomes coarse, and the Ni_2_AlHf phase becomes fine and uniformly distributed. The heat treatment mainly optimizes the size and distribution of Ni_2_Al_3_Ho and Ni_2_AlHf phases in the NiAl-Cr(Mo)-Hf-Ho eutectic alloy.
(3) The heat treatment helps to eliminate interface dislocations in the large NiAl precipitates and along the NiAl/Cr(Mo) phase interface, which also contributes to the fracture toughness by decreasing the stress concentration.
(4) Compared with the as-cast NiAl-Cr(Mo)-Hf eutectic alloy, the minor Ho addition decreases the fracture toughness from 6.7 to 6.1 MPa·m^1/2^, which should be mainly ascribed to the coarsened intercellular zone including Ni_2_Al_3_Ho and Ni_2_AlHf phases. The heat treatment increases the fracture toughness of NiAl-Cr(Mo)-Hf-Ho eutectic alloy from 6.1 to 8.2 MPa·m^1/2^, which is also higher than that of the heat-treated NiAl-Cr(Mo)-Hf eutectic alloy. The improved fracture toughness of heat-treated NiAl-Cr(Mo)-Hf-Ho eutectic alloy should be ascribed to the refined and well-distributed Ni_2_Al_3_Ho and Ni_2_AlHf phases.

## Figures and Tables

**Table 1:** Chemical composition of constituent phases in as-cast NiAl-Cr(Mo)-Hf and NiAl-Cr(Mo)-Hf eutectic alloys.

Alloy	Phase	Ni	Al	Cr	Mo	Hf	Ho
As-cast NiAl-Cr(Mo)-Hf	NiAl	47.06	49.23	3.42	0.08	0.21	—
	Cr(Mo)	6.76	5.49	70.92	16.83	—	—
	Ni_2_AlHf	47.64	27.82	1.49	0.06	22.99	—
As-cast NiAl-Cr(Mo)-Hf-Ho	NiAl	51.38	46.31	2.04	—	0.27	—
	Cr(Mo)	4.95	8.04	76.93	10.08	—	—
	Ni_2_AlHf	51.77	25.32	2.62	—	18.53	1.76
	Ni_2_Al_3_Ho	33.64	45.21	1.96	—	1.16	18.03

## Data Availability

Not applicable.
